# Distribution of Gadolinium in Rat Heart Studied by Fast Field Cycling Relaxometry and Imaging SIMS

**DOI:** 10.3390/ijms20061339

**Published:** 2019-03-16

**Authors:** Claudia Bonechi, Marco Consumi, Marco Matteucci, Gabriella Tamasi, Alessandro Donati, Gemma Leone, Luca Menichetti, Claudia Kusmic, Claudio Rossi, Agnese Magnani

**Affiliations:** 1 Department of Biotechnology, Chemistry and Pharmacy, Via Aldo Moro 2, 53100 Siena, Italy; marco.consumi@unisi.it (M.C.); gabriella.tamasi@unisi.it (G.T.); gemma.leone@unisi.it (G.L.); claudio.rossi@unisi.it (C.R.); 2 Center for Colloids and Surface Science (CSGI), Via della Lastruccia 3, 50019 Sesto Fiorentino, Firenze, Italy; 3 National Interuniversity Consortium of Materials Science and Technology (INSTM), Via Giuseppe Giusti 9, 50121 Firenze, Italy; 4 CNR Institute of Clinical Physiology, Area di Ricerca “S. Cataldo”, Via Giuseppe Moruzzi 1, 56124 Pisa, Italy; matteucci@ifc.cnr.it (M.M.); luca.menichetti@ifc.cnr.it (L.M.); kusmic@ifc.cnr.it (C.K.)

**Keywords:** NMRD profiles, gadolinium, ToF-SIMS, tissue microimaging

## Abstract

Research on microcirculatory alterations in human heart disease is essential to understand the genesis of myocardial contractile dysfunction and its evolution towards heart failure. The use of contrast agents in magnetic resonance imaging is an important tool in medical diagnostics related to this dysfunction. Contrast agents significantly improve the imaging by enhancing the nuclear magnetic relaxation rates of water protons in the tissues where they are distributed. Gadolinium complexes are widely employed in clinical practice due to their high magnetic moment and relatively long electronic relaxation time. In this study, the behavior of gadolinium ion as a contrast agent was investigated by two complementary methods, relaxometry and secondary ion mass spectrometry. The study examined the distribution of blood flow within the microvascular network in *ex vivo Langendorff* isolated rat heart models, perfused with Omniscan^®^ contrast agent. The combined use of secondary ion mass spectrometry and relaxometry allowed for both a qualitative mapping of agent distribution as well as the quantification of gadolinium ion concentration and persistence. This combination of a chemical mapping and temporal analysis of the molar concentration of gadolinium ion in heart tissue allows for new insights on the biomolecular mechanisms underlying the microcirculatory alterations in heart disease.

## 1. Introduction

Coronary microcirculation plays an important role in human pathology. Research on the biomolecular mechanisms that lead to microcirculatory alterations related to human cardiopathies is essential to understand the genesis of myocardial contractile dysfunction and coronary atherosclerotic disease. New approaches are needed to identify, prevent, and treat these conditions. This requires analytical technologies based on specific biomarkers or the investigation of biological tissues areas of small or very small dimensions with high sensitivity and high lateral resolution. 

The use of complementary approaches to the study of the mechanisms that control the distribution of the blood flow within the microvascular network presents several advantages. The synergic use of mass spectrometry and relaxometric experiments allows for both mapping and quantification of the intracellular content of flow tracers. Such information presents multiple advantages to improving our understanding of the physio-pathological mechanisms that lie at the base of an improved prevention and treatment. 

Time of flight secondary ion mass spectrometry (ToF-SIMS), with its high sensitivity (in the order of ppm), provides a chemical map with a submicron lateral resolution [1]. The extreme sensibility of this technique to the sample surface makes sample preparation critical to preserving the native structure and chemistry of biological tissues. Freeze-dried and frozen-hydrated approaches are the main routes to analyze cultured cells and biological samples [2]. Biological tissues could lose their biomarker distribution if chemical fixation treatments are applied. By avoiding chemical fixation, ToF-SIMS analysis of biomarked tissues provides significant advantages and has been successfully used in the study of both surfaces and thin coatings [3,4,5,6,7], polymeric and biological samples [8,9,10], as well as biomarkers and cellular component distribution [11,12,13,14,15]. ToF-SIMS was widely used to obtain images of biological materials and tissues, like heart, brain, liver, etc.; many works can be found in the literature [16,17,18].

Fast field cycling (FFC) relaxometry is a low-resolution technique measuring nuclear longitudinal relaxation rates as a function of the magnetic fields, from 0.01 to hundreds of MHz [19]. The spectral density function of the observed nuclei (typically the solvent water protons) can be directly accessed for the study of contrast agents for magnetic resonance imaging [20,21,22,23]. Relaxometry has been used to detect the collective relaxation rate of protein protons, thus obtaining direct information on their spectral density function [24]. Relaxometry has been typically used to study new materials, while its use in biomedical fields are very limited. 

There is a growing need to develop synergistic approaches to characterize metal-based drugs [25,26,27,28,29] and identify biomarkers for the assessment of the interaction [30] and inclusion of molecules with target tissues [31,32,33]. Numerous studies have shown the advantage of combining spectroscopy, diffractometry, and theoretical methods [34,35,36,37,38]. NMR studies have often been used to examine the ligand–receptor processes [39,40,41,42] and water–protein interactions [43]. However, compared to NMR spectroscopy and imaging (MRI), relaxometry has not been well explored.

Gadolinium compounds are used as diagnostic and theranostic agents [44,45,46]. Gadolinium contrast agents have been used to detect amyloid β-protein aggregates by MRI [47]. However, gadolinium accumulates in different human body regions, particularly in the brain, following repeated use of gadolinium-based contrast agent (GBCA) therapy [48,49,50]. 

In the present study, the simultaneous application of relaxometry and ToF-SIMS for the study of the microperfusion of the heart was explored in terms of both quantification (relaxometry) and blood flow mapping at high resolution (microns) of coronary micro-circulation. This was achieved by mapping the distribution of the flow tracer within the microvascular network in *ex vivo Langendorff* isolated rat heart models under different perfusion protocols that reproduced physiological and low-flow ischemic conditions.

## 2. Results and Discussion

### 2.1. Fast Field Cycling Relaxometry Fast field cycling relaxometry

Fast field cycling relaxometry experiments on homogenized rat heart tissue perfused with Omniscan were performed to check the presence of gadolinium in the heart tissue and to confirm its persistence. The first phase of the study involved the rat heart tissue homogenized with and without the addition of contrast agent. The pattern of relaxation rate (R_1_) versus magnetic field was strongly influenced by the presence of Gd(III), and, in particular, the ability of the Gd(III)-chelate to enhance the water proton nuclear magnetic relaxation rates was related to its paramagnetic properties. 

Comparing the experimental results on homogenized tissue with and without the addition of contrast agent, the presence of Gd(III) was confirmed by obtaining values of relaxation rates (R_1_ = 7.15 ± 0.40 s^−1^) that were significantly higher than those of the reference samples (R_1_ = 0.62 ± 0.05 s^−1^). This result was also obtained for the different perfusates.

**Figure 1:**
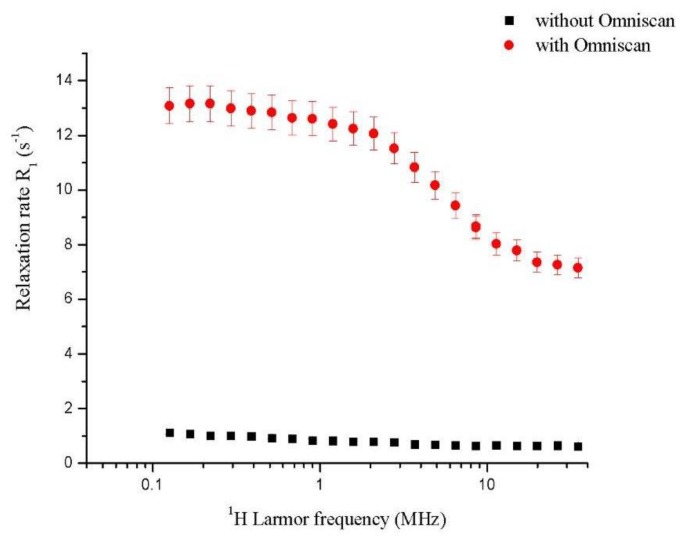
^1^H-NMRD profile for homogenate rat heart: Without perfusion of Omniscan and with perfusion of Omniscan. For all relaxation rate values, the error bar (5%) was reported.

The dispersion curves showed significant differences with and without Gd(III) (Figure 1). The homogenized heart tissue without Gd(III) brought about an NMRD curve that followed a power function, typical of immobilized proteins [19,51] and biological tissues. Previously published studies have shown that the proton spin-lattice relaxation rate constants in immobilized proteins follow a power function that can be related to a spin-phonon-like relaxation mechanism [52]. Figure 1 reports the NMRD profile of homogenized tissue after perfusion with Gd(III), which showed a classical lorenztian shape, characteristic of Gd(III) complexes [53]. In this case, the influence by paramagnetic species on the relaxation processes (R_1_) followed a typical mathematical function reported in a previous paper [54]. This experimental result confirmed that, in the homogenized heart tissue treated with Omniscan, the gadolinium was persistent and had strong influence in the relaxation phenomena [55,56].

The second stage of the study was relevant to determine the concentration of Gd(III) in the perfusate and homogenized heart tissue. The protocol that was optimized was based on the use of the standard additions method to allow a better control of the matrix effect. Indeed, the standard additions method is commonly used to determine the concentration of selected analytes in complex matrixes, such as biological fluids, soil samples, etc. This approach was used to avoid the interference of other components with the analyte signal. The procedure for standard additions involved the addition of small amounts of gadolinium standard solution in the samples under study (homogenized heart tissue), then the longitudinal water proton relaxation rate was measured for all of the samples and the data were plotted versus molar concentration of added gadolinium. Linear regression was calculated and the slope and intercept of the calibration curve were used to calculate the concentration of gadolinium in the sample under study.

**Figure 2:**
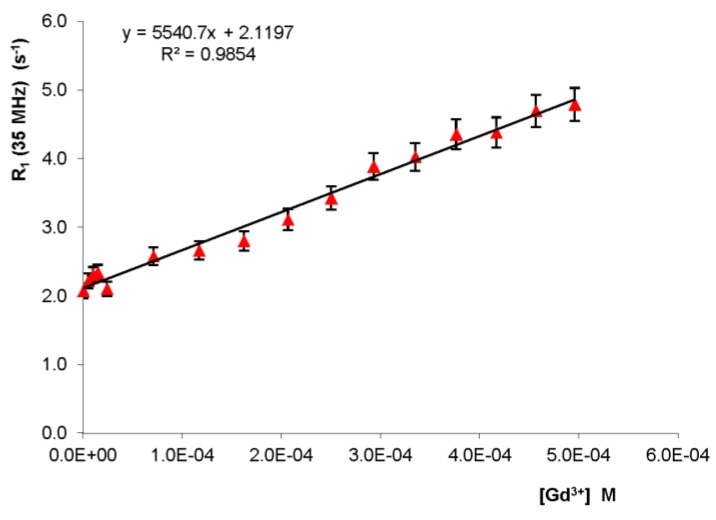
^1^H NMR relaxation rate (R_1_) of a rat heart homogenized with standard addition of Gd^3+^ and linear fitting.

Interestingly, the increase of the water proton relaxation rate (i.e., R_1_) of a homogenized heart tissue measured at 35 MHz proton Larmor frequency was found to be a linear function of the gadolinium concentration at 298 K (Figure 2). 

The linear dependence had a slope of 5541 ± 275 M^−1^·s^−1^ (R^2^ = 0.985). The concentration of paramagnetic ions in the homogenized tissue of the rat heart with Omniscan perfusion was determined through the standard addition method [57]; setting the y-intercept to 0, the molar concentration Gd(III) was calculated as 0.385 ± 0.019 mM.

However, this value also included the paramagnetic contribution of all species present in the heart, regardless of the perfusion of Omniscan. To eliminate this interference, relaxometry experiments were performed on the rat heart which was not perfused by the marker. The resulting water relaxation rate was 0.62 s^−1^. Using this value of R_1_ as the y-intercept for a straight line parallel to that of Figure 2, the molar concentration of paramagnetic species in the heart not perfused was 0.113 ± 0.010 mM.

The molar concentration of Gd(III) due to perfusion of rat heart with the *Langendorff* procedure was the difference between the two molar concentrations (0.385 ± 0.019 mM and 0.113± 0.010 mM), so Gd(III) = 0.272 ± 0.029 mM. 

### 2.2. Analysis of Heart Tissue Sections

#### 2.2.1. Optical Analysis 

**Figure 3:**
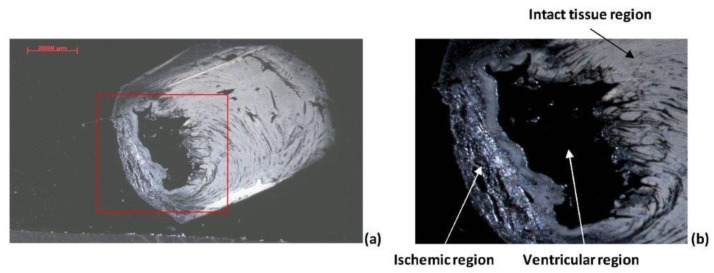
Optical image of (**a**) the full heart tissue section, and (**b**) the magnification of the region close to the ventricle showing the damaged tissue (ischemic area).

Figure 3 shows the optical image of the infracted heart tissue section. Three main regions can be distinguished: i) ventricular region; ii) ischemic region; iii) intact tissue region.

As evidenced by the magnification of the area close to the ventricle (Figure 3b), ischemic and non-ischemic tissues show some significant differences. The non-ischemic region appears smooth and compact. The ischemic (damaged) region is instead characterized by a more “spongy” tissue full of pores. In this region, cells are fibrous and the area appears the least compact zone of the whole section. 

#### 2.2.2. ToF-SIMS Analysis of Heart Tissue Sections: Chemical Maps of Gd(III) Distribution

SIMS is a surface technique that has been successfully used to probe cells and tissues over the last few decades. As with most mass spectrometric approaches, the details of sample preparation can often determine the success of a measurement. Due to the higher spatial resolution, analyte redistribution issues can become critical. Ischemic and physiological heart tissue sections were investigated to obtain a chemical map of the flow tracer distribution within the microvascular network under ischemic or physiological conditions, which is useful for the assessment of coronary microcirculation. 

The focus of this study was also to preserve, by the optimization of the sample preparation process, the fine structure of hearth tissue to be imaged. Multiple samples were prepared and imaged to verify that the analyte redistribution is not related to sample preparation.

#### 2.2.3. Physiological Heart Tissue Sections

**Figure 4:**
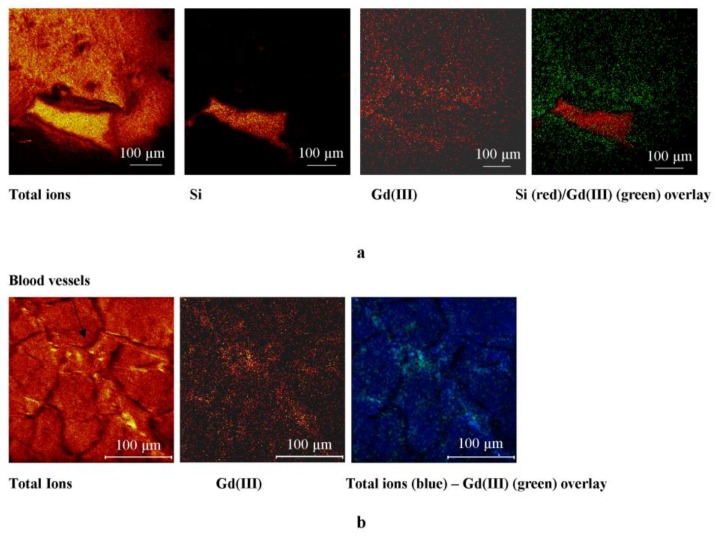
ToF-SIMS maps of non-ischemic tissue: (**a**) Heart tissue section close to the ventricle (image area: 700 × 700 µm); (**b**) heart tissue section near blood vessels (image area: 250 × 250 µm).

Figure 4 shows the distribution of Gadolinium in the most representative region of the non-ischemic sample. The whole section shows a higher amount of the marker close to the ventricle and near the blood vessels. 

#### 2.2.4. Ischemic Heart Tissue Sections

**Figure 5:**
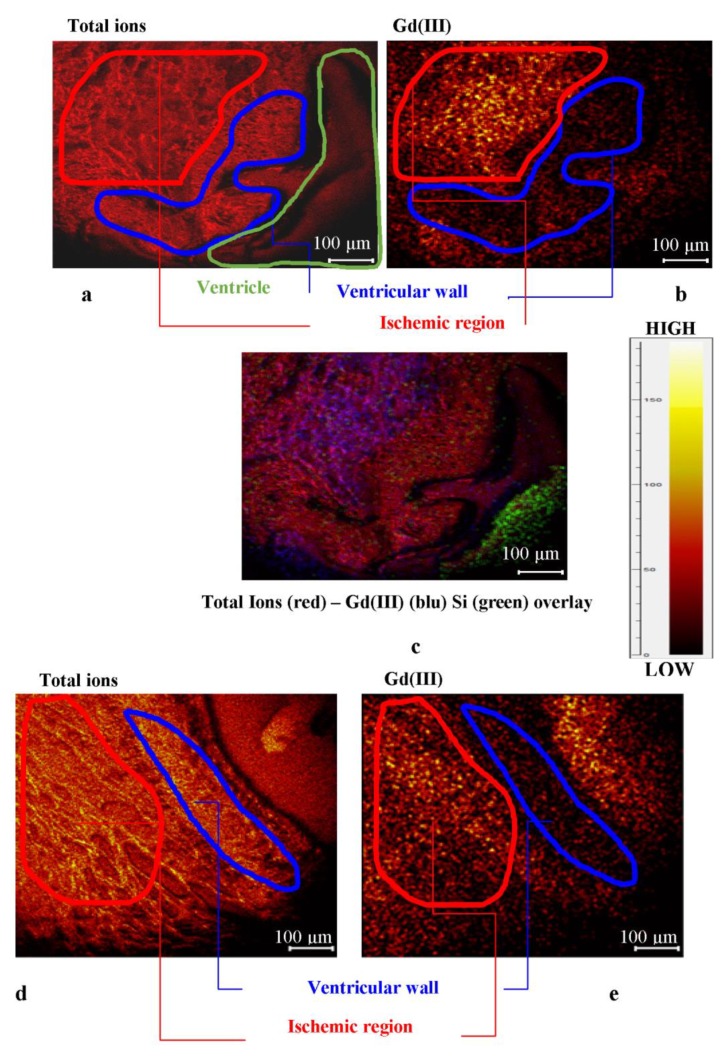
(**a**) Total ions image that highlights three different areas of the sample: Ischemic region (upper left), ventricular wall (middle), and ventricle (lower right); (**b**) distribution map of Gd(III); the image shows accumulation in the ischemic region (upper left); (**c**) ion distribution overlay with Gd (blue) and Si (green); (**d**) total ions image of a different region of the sample; (**e**) Gd(III) distribution map highlighting the accumulation of the tracers in the ischemic region beyond the ventricular wall.

Figure 5 shows the distribution of the marker in the most representative region of the ischemic samples. The optical image (see Figure 3) showed some differences in the ischemic and non-ischemic regions within the area of the heart tissue section close to the ventricle. According the optical analysis, the SIMS study highlighted different marker distribution between the ischemic and non-ischemic regions. Gd(III) accumulated within the ischemic region, showing a lower intensity signal in the intact ventricular wall.

The map of the biomarker in the most representative region within the physiologic heart section shows a distribution gradient with accumulation on the ventricle, the vessel wall, or close to them. On the contrary, the infarcted heart section shows a Gd accumulation within the ischemic region. In the healing area, fibrous tissue replaced the necrotic tissue; this process is associated with a significant expansion of the interstitial space and a subsequent increase in the volume of distribution of gadolinium.

## 3. Materials and Methods

**Figure 6:**
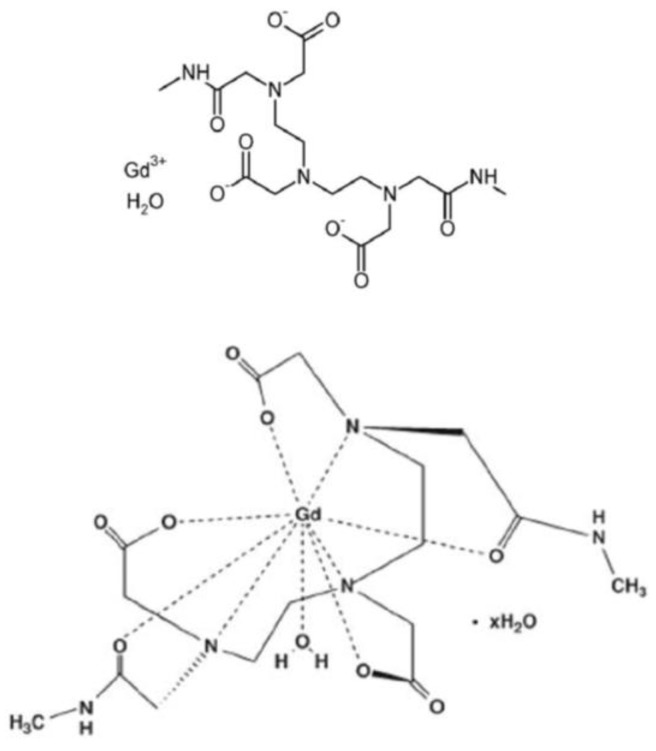
Structure of Omniscan, Gd(DTPA-BMA)(H_2_O).

The MRI contrast agent used in this study was Omniscan^®^ (Amersham Health, Princeton, NJ, USA; [Gd(DTPA-BMA) (H_2_O)], where DTPA-BMA is 1,7-bis[(*N*-ethylcarbonyl)methyl]-1,4,7-triazaheptane-1,4,7-triacetic acid (Figure 6)), a gadolinium-based contrast agent (GBCA) containing 287 mg/mL of gadodiamide (537 MW). 

### 3.1. Animal Models and Isolated Heart Preparation

Male Wistar rats (Envigo, Udine, Italy) of 300–350 g and 12–14 weeks of age were used. Isolated hearts of healthy rats and rats with left ventricular anterior wall infarction at 14 days from the permanent ligation of the left anterior descending coronary artery were studied. For isolated heart preparation, animals were heparinized (500 U i.m.) 10 min prior to anesthesia with pentobarbitone sodium (40 mg/kg, i.p.). Hearts were excised and placed in ice-cold Krebs–Henseleit bicarbonate solution (KHB) of the following composition: NaCl 118 mM, NaHCO_3_ 24 mM, KCl 4.7 mM, KH_2_PO_4_ 1.2 mM, MgSO_4_ 1.2 mM, CaCl_2_ 2.5 mM, EDTA 0.5 mM, and glucose 5.5 mM. The solution was pre-equilibrated with 95% O_2_ and 5% CO_2_ at pH 7.4 ± 0.1. After removal of foreign tissues, the aorta was cannulated and the heart transferred into a non-recirculating *Langendorff* apparatus, where it was allowed to beat spontaneously and retrogradely perfused at a constant pressure by 70 mmHg with oxygenated KHB (95% O_2_–5% CO_2_ mixture). The hearts were maintained at 37 °C by a thermostatically controlled chamber and coronary flow was continuously measured with a flowmeter (model T106, Transonic System Inc., Ithaca, NY, USA) coupled with an in-line flow probe. Portions of perfusate were also collected for comparison reasons and furthers analyses.

After a 30-min period of perfusion with KBH solution, the non-radioactive tracer (Gadolinium(III) chelate compound, Omniscan) was infused into continuous mode through a side arm in the perfusion line. At the third minute of Omniscan infusion the heart was quickly detached from the cannula and immediately frozen in isopentane pre-cooled in liquid nitrogen (−150 °C) and stored at –80°C. It can be estimated that the Omniscan solution achieved the concentration range of 60–100 mM in the line of perfusion and then at the entrance of the coronary tree. As a time of infusion of the extracellular contrast agent, a value was chosen that was double/triple the time to maximum relative enhancement reported for tissue signal enhancement in MRI studies on rats injected with a bolus of Gd [58,59].

### 3.2. Sample Preparation 

The whole heart was excised and ventricles were weighted. A mid-ventricular transversal slice about 2 mm thick was cut, frozen in liquid nitrogen, and stored for relaxometry analysis. The remaining portion of the ventricles were rapidly frozen in isopentane cooled at −150 °C in liquid nitrogen and stored to be processed for ToF–SIMS analysis. 

For ToF-SIMS experiments, coronal sections 8–20 μm thick were obtained using a cryo-microtome. Cutting operations were carried out at −25 °C. The sections were then adhered onto a silicon substrate, taking extreme care in preventing them from defrosting. The obtained sections were freeze-fractured by accommodating the coronal sections in a freeze-etch unit (Balzers 301) and fracturing them at about −115 °C. Then, the samples were warmed to −80 °C and the metal knife was left over the samples as a cold shroud for 60 minutes, drying at −80 °C. The samples were then allowed to reach room temperature and analyzed without further treatment. The major disadvantage of the freeze-fracturing method is that it requires a long time for the preparation of a single sample.

For relaxometric measurements the perfused rat heart was homogenized. 

### 3.3. Optical Microscopy Measurements

Optical micrographs of heart tissue sections were obtained by a stereo-microscope Olympus, Germany, equipped with an Axiocam MRC5, Zeiss, Germany.

### 3.4. ToF-SIMS Measurements

ToF-SIMS measurements were carried out on a TRIFT III spectrometer (Physical Electronics, Chanhassen, MN, USA) equipped with a gold liquid–metal primary ion source. Before acquiring positive and negative spectra the samples were maintained overnight in a conditioning pre-chamber with a vacuum value of about 10^−4^ Pa and then moved to the analyzing chamber in which the vacuum value was raised up to 10^−8^ Pa. Positive and negative ion spectra were acquired with a pulsed, bunched 22 keV Au^+^ primary ion beam by rastering the ion beam over a predefined sample area and maintaining static SIMS conditions (primary ion dose density 10^12^ ions/cm^2^). Positive ion spectra were calibrated with CH_3_^+^ (*m*/*z* 15.023), C_2_H_3_^+^ (*m*/*z* 27.023), and C_3_H_5_^+^ (*m*/*z* 41.039) and negative ion spectra were calibrated with CH^−^ (*m*/*z* 13.008), OH^−^ (*m*/*z* 17.003), and C_2_H^−^ (*m*/*z* 25.008), in the low mass region and with I^−^ (m/z 126.90) in the high mass region. A number of peaks of increasing mass were assigned and added to the calibration set for an accurate mass calibration. The mass resolution (m/Δm) was 6000 at *m*/*z* 27. Chemical images were acquired with a pulsed, unbunched 22 keV Au^+^ primary ion beam by rastering the ion beam over a predefined sample area sample area and maintaining static SIMS conditions. The lateral resolution was about 1 micron. 

To evaluate the tracer distribution, each section has been divided into various analysis areas: a) Portion of the right ventricular wall, b) portion of the posterior wall of the left ventricle, c) portion of the anterior wall of the left ventricle, and d) portion of the interventricular septum. The sections to be analyzed at SIMS were previously observed by scanning electron microscope (SEM) in order to obtain a precise anatomical reference and morphology for SIMS imaging.

### 3.5. Relaxation Rate Measurements

The samples for ^1^H-NMRD experiments were prepared using mid-ventricular cardiac tissue harvested and stored for this analysis. Briefly, the frozen heart slice was pulverized in liquid nitrogen and 130 mg of tissue (about 10% of the total ventricle weight) was homogenized in cold phosphate buffer (PBS, 50 mM; pH 7.4). 

Longitudinal water proton relaxation rates were measured with a Stelar fast field cycling relaxometer (0.01–40 MHz proton Larmor frequency range). The instrument provided R_1_ values with an error smaller than 3–5%, as reported also by Foster et al. 2016 [60]. ^1^H-NMRD profiles were obtained by plotting proton relaxation rates as a function of applied magnetic field. In the experimental magnetization trend, the error bars on R_1_ values was reported with a value of 5%.

Water proton T_1_ measurements at 0.02 MHz were recorded on a Stelar Spinmaster-FFC field cycling NMR relaxometer by measuring the magnetization decay at a field strength of 4.7310 T (corresponding to 0.02 MHz proton Larmor frequency) after a pre-polarization period at 0.22 T. Temperature (25.0 ± 0.5 °C) was controlled by a Stelar VTC-91 airflow heater, equipped with a copper constantan thermocouple.

### 3.6. Determination of Gadolinium Content in Homogenized ex vivo Langendorff Isolated Rat Hearts

The analytical determination of Gd(III) content in the heart samples was performed via the standard additions method. In fact, this method permits to overcome the matrix effect of biological samples. First the sample “as it is” was measured and then the sample was spiked by known amounts of Gd(III) standard solution (0.5 mM 10 L, Sigma). Fifteen additions for each sample were performed. The dilution effect was considered in the calibration curves; those showing correlation factors R^2^ > 0.980 were accepted for analyses. The results were expressed as mM Gd(III).

For each homogenized rat heart tissue, three samples were prepared and for each sample measurements were carried out in triplicate.

### 3.7. Statistical Data Treatment

All samples were analyzed in triplicate and mean values and estimated standard deviations were calculated and reported. Calculation were carried out by using Microsoft Office Excel 2007 implemented with regression analysis subroutine and Origin Pro8 SR2, v.0891 (B891).

## 4. Conclusions

This paper demonstrated the potential of relaxometry and imaging ToF-SIMS as useful tools in biomedical research for the assessment of coronary microcirculation in small organs like murine hearts. In particular, the new relaxometric experimental procedure allowed the determination of the concentration of paramagnetic tracers such as gadolinium-based biomarkers (gadodiamide) used in imaging techniques. Relaxometry allowed the analysis of different behaviors between homogenized heart tissue untreated and treated with gadolinium. This procedure permitted an easy and accurate determination of the concentration of Gd(III) perfused in heart using the *Langendorff* equipment.

The imaging ToF-SIMS provided useful data for understanding the effect of ischemic events through the analysis of the gadolinium-based flow tracer (gadodiamide) distribution within ischemic and non-ischemic areas of the myocardial tissue. The map of the biomarker in the most representative region within the non-ischemic heart section showed a distribution gradient with accumulation on the ventricle, the vessel wall, or close to them. On the contrary, the ischemic heart section evidenced a Gd accumulation within the damaged region, demonstrating that the ischemic event is associated with an accumulation of the biomarker within the compromised tissue. Further analysis of the diseased regions could be used to improve tissue-based diagnosis in combination with gadolinium-based biomarkers.
